# Cohort profile: Korean Varicella Immunization Monitoring (K-VIM) Scheme: a national cohort of children born 2011–2022

**DOI:** 10.4178/epih.e2026005

**Published:** 2026-01-22

**Authors:** Young Kyu Shim, Young Hwa Lee, Young June Choe, Yoonsun Yoon, Yun-Kyung Kim

**Affiliations:** Department of Pediatrics, Korea University College of Medicine, Seoul, Korea

**Keywords:** Chickenpox, Chickenpox vaccine, Vaccination, Republic of Korea

## Abstract

Varicella, caused by the varicella-zoster virus, was once nearly universal in childhood before the advent of vaccination and may lead to severe complications and even fatalities. Monitoring varicella vaccine effectiveness is crucial yet often overlooked in settings with limited surveillance infrastructure. The Korean Varicella Immunization Monitoring (K-VIM) Scheme was established to address this gap by assembling a national, insurance-based birth cohort of children born between 2011 and 2022 (n=4,505,165). This cohort leverages comprehensive healthcare databases in Korea to capture vaccination records, medical visits, and varicella infection outcomes for virtually all children within the target birth years. We describe the enrollment and key characteristics of the K-VIM cohort, including vaccination coverage, demographic features, and varicella incidence to date. The structure and completeness of Korea’s national health insurance and immunization registries enable robust longitudinal tracking of varicella infections among vaccinated versus unvaccinated children. Early findings demonstrate distinct patterns in infection rates and disease severity according to vaccination status. The K-VIM Scheme provides a foundation for ongoing epidemiological studies of varicella vaccine effectiveness and public health impact in Korea. Future plans include evaluating the long-term effects of varicella vaccination, including potential impacts on herpes zoster incidence, using diverse study designs to strengthen causal inference and inform immunization policy.

## INTRODUCTION

Varicella, caused by the varicella-zoster virus (VZV), is characterized by a distinctive vesicular rash and was once widespread in childhood prior to the introduction of vaccination. After primary infection, VZV can remain latent in sensory nerve ganglia and may later reactivate as herpes zoster [1]. Although varicella is often regarded as a mild childhood illness, it can result in serious complications, including fatalities, particularly among vulnerable populations such as immunocompromised individuals and infants [2].

Since the introduction of the live-attenuated varicella vaccine, both the incidence of varicella and varicella-related mortality have declined substantially. A case-control study conducted in the United States between 1997 and 2000 found the vaccine to be 85% effective against polymerase chain reaction-confirmed VZV infection and 97% effective against moderate to severe disease [3]. In addition, an analysis of national death records from 1990 to 2001 demonstrated a marked reduction in varicella-related mortality, with death rates decreasing by 66% from 1990–1994 to 1999–2001. This decline was most pronounced among children aged 1 year to 4 years, underscoring the effectiveness of childhood varicella vaccination programs in reducing varicella-associated deaths [4].

Despite these successes, a critical gap remains in the monitoring of varicella vaccine effectiveness in children, particularly in countries with limited disease surveillance capacity [5]. In many settings, insufficient resources are allocated to systematically assess the incidence of vaccine-preventable diseases, despite the central role such evaluations play in confirming vaccines’ public health impact [6]. As a result, the capacity to rigorously evaluate vaccine effectiveness often lags behind vaccine introduction, leading to uncertainty regarding real-world vaccine performance and population-level impact.

Parallels can be drawn with other large-scale vaccine effectiveness initiatives, such as those for influenza and coronavirus disease 2019 (COVID-19), which highlight the value of dedicated monitoring efforts. The Flu Watch study, established to examine multiple dimensions of influenza vaccine effectiveness, provides a useful model for comprehensive evaluation within a defined population [7]. Similarly, the Danish National Cohort Study of Effectiveness and Safety of SARS-CoV-2 vaccines recruited birth cohorts as well as older children and adults to investigate vaccine effectiveness, impact, coverage, safety, and indirect effects of COVID-19 vaccination [8].

In Korea, despite high coverage with a single dose of varicella vaccine, outbreaks of varicella continue to occur. Several studies have suggested substantial waning of vaccine-induced immunity over time following a single-dose regimen. In this context, ongoing large-scale monitoring is essential to assess the long-term effectiveness of the current National Immunization Program (NIP) and to evaluate the real-world impact of 1-dose versus 2-dose vaccination schedules. In response to this need, the Korean Varicella Immunization Monitoring (K-VIM) Scheme was established as a national, insurance-based cohort of children born between 2011 and 2022. This initiative is designed to generate comprehensive evidence on the effectiveness, safety, and coverage of varicella vaccination among Korean children. By leveraging a robust national infrastructure for data collection and analysis, similar to that used in previous vaccine effectiveness studies, the K-VIM Scheme enables continuous monitoring of varicella vaccination outcomes and supports informed decision-making in public health policy and practice.

In this cohort profile, we describe the study population, data sources, and data management processes of the K-VIM Scheme, and we highlight its potential to serve as a cornerstone for ongoing epidemiological research on varicella vaccine effectiveness in Korea.

## COHORT DESCRIPTION

### Setting

In Korea, the National Health Insurance system provides healthcare coverage to all citizens, with funding primarily derived from contributions by insured individuals and government subsidies [9]. The system comprises 2 core institutions: the National Health Insurance Service (NHIS), which functions as the insurer, and the Health Insurance Review and Assessment Service (HIRA), which is responsible for claims review and quality assessment of healthcare services [10]. Healthcare providers submit reimbursement claims to HIRA through the web-based Medical Claim Portal Service. HIRA employs an information technology system to perform initial error checks and conducts electronic claim reviews based on predefined criteria [11]. Claims identified as outliers are further examined by HIRA staff, medical professionals, or expert review committees. Upon completion of the claims review process, HIRA forwards the finalized results to the NHIS for provider reimbursement.

The NIP for children is a central public health initiative that provides immunization services against infectious diseases in Korea [12]. The program fully covers the cost of vaccinations against 17 essential vaccine-preventable diseases, as defined by the Korea Disease Control and Prevention Agency (KDCA) NIP guidelines during the study period. By offering cost-free immunization services through designated healthcare clinics, the NIP has achieved high vaccine coverage and promotes herd protection at the population level. All children under 12 years of age are eligible beneficiaries of the NIP. The program provides full coverage for all government-designated vaccines, encompassing a total of 17 recommended immunizations [13]. These vaccines include, but are not limited to, Bacillus Calmette–Guérin (percutaneous), hepatitis B, diphtheria–tetanus–acellular pertussis, tetanus–diphtheria, tetanus–diphtheria–acellular pertussis, inactivated poliovirus vaccine, and measles–mumps–rubella [14]. Several imported and domestically produced varicella vaccines have entered the market at different time points. As varicella vaccine coverage increased nationwide, varicella was designated a nationally notifiable disease. In 2005, the varicella vaccine was incorporated into the NIP and mandated for universal immunization at government expense, with routine vaccination of infants aged 12 months to 15 months strongly recommended. To strengthen surveillance following implementation of the program, varicella was formally designated as a nationally notifiable disease in July 2005.

All individuals residing in Korea are assigned a unique personal identification code. Korea maintains extensive national registers containing health, demographic, and socioeconomic information collected for administrative purposes and linked at the individual level using this personal identification code. These data are collected automatically, thereby minimizing systematic reporting biases such as recall bias. All register information is time-stamped, allowing exposures and outcomes to be temporally linked and enabling investigation of the cumulative effects of immunization on varicella incidence. Accordingly, the structure of the Korean registry system provides a unique opportunity to evaluate the real-world effectiveness of varicella vaccination while accounting for a wide range of potential confounding factors.

### Source and content of data

Varicella vaccination records for each child were obtained from the National Immunization Registry, which is maintained by the KDCA. Healthcare providers are required to report all NIP vaccine doses administered, typically in real time through a web-based reporting system. From this registry, we extracted the dates of varicella vaccinations, the dose number, and the specific vaccine product administered for each child. Owing to the mandatory reporting requirement, registry data for this cohort are nearly complete, and missing or unknown vaccine information was rare.

Varicella infection events were identified using HIRA’s nationwide health insurance claims database. We retrieved all insurance claims containing a varicella diagnostic code (International Classification of Diseases, 10th revision [ICD-10] code B01.x) for cohort children, regardless of whether care was provided in outpatient or inpatient settings. In Korea, varicella is a nationally notifiable disease, and most cases are diagnosed based on clinical presentation; laboratory confirmation is not routinely required for notification. Claims data provide information on the dates of healthcare encounters and whether cases were managed in outpatient clinics or hospitalized settings. We also obtained data on antiviral treatments prescribed, although treatment variables are not the primary focus of this cohort profile. Medication information in HIRA claims is coded using the Anatomical Therapeutic Chemical classification system, which may be leveraged in future analyses to infer disease severity, such as whether antiviral therapy was administered.

All datasets were linked at the individual level using the unique personal identification code. To protect privacy, analyses were conducted using de-identified data. Data linkage and de-identification procedures were performed within a secure government data center environment, and researchers accessed only a pseudonymized, non-linkable dataset. Analytical outputs were aggregated to cell sizes exceeding minimum disclosure thresholds to prevent potential re-identification. Because the cohort relies exclusively on pre-existing administrative data, no direct contact with study participants was required.

### Study population

The K-VIM cohort includes all children born between January 1, 2011, and December 31, 2022, in Korea, as well as children born abroad during these years who subsequently immigrated to Korea and obtained residency between 2011 and 2022 (Table 1). The cohort is defined strictly by birth year; individuals born before 2011 were not included, regardless of the timing of immigration. Using linked data from the National Immunization Registry and HIRA claims database, we identified approximately 4.53 million eligible children across these birth cohorts. After excluding a small number of children with uncertain residency status or missing key information, the final analytic cohort comprised 4,505,165 children. This population represents virtually all children born during the 2011–2022 period nationwide. During this interval, annual births in Korea declined substantially, from approximately 480,000 in 2011–2012 to about 250,000 in 2022, reflecting the country’s sustained decrease in birth rates. Table 1 presents cohort enrollment stratified by birth year and sex. Overall, cohort members were 51.3% male and 48.7% female, consistent with the slight male predominance observed in annual live births. Children entered the cohort at birth and were followed prospectively from that time forward. For this cohort profile, follow-up data were available through December 31, 2022. Children contributed person-time until their last recorded healthcare encounter or until administrative censoring at the end of the study period. By linking immunization registry data with insurance claims, each child’s varicella vaccination history and subsequent varicella diagnoses could be longitudinally ascertained.

#### Characteristics of the study population

Within the K-VIM cohort, virtually all children received at least 1-dose of varicella vaccine through the routine immunization program; however, a subset received a second dose, and a small minority remained unvaccinated as of the end of follow-up. In total, 298,533 children (6.6%) had not received any varicella vaccine, 2,852,547 children (63.3%) had received 1-dose, and 1,321,737 children (29.3%) had received 2-dose of varicella vaccine. Table 2 compares demographic and vaccination-related characteristics across these 3 groups (unvaccinated, 1-dose, and 2-dose). As shown in Table 2, the unvaccinated group is dominated by children born in 2022, who comprise 61.9% of this group. This pattern reflects the study follow-up ending on December 31, 2022, at which time children in the 2022 birth cohort were all younger than 12 months and had not yet reached the NIP-recommended vaccination age of 12–15 months. These children are therefore classified as unvaccinated due to age ineligibility rather than vaccine refusal. Children’s varicella vaccination status is closely linked to birth cohort, reflecting evolving recommendations and differential opportunities to receive additional doses over time. Accordingly, significant differences in birth-year distribution were observed among the vaccination groups (p<0.001). It is also important to note that the Korean NIP funds only a 1-dose varicella vaccination schedule. The substantial proportion of children who received a second dose (29.3%) therefore reflects privately funded, out-of-pocket vaccination. In response to continued breakthrough infections, many pediatricians recommend a second dose, typically administered at 4–6 years of age, which parents obtain through private payment.

Aside from differences in birth-year distribution, the vaccination groups were similar with respect to demographic characteristics. Sex distribution was approximately equal across unvaccinated, 1-dose, and 2-dose children, with a slight male predominance in all groups, consistent with the national sex ratio at birth. The mean age of children at the end of follow-up varied by birth cohort, ranging from infants to 11-year-olds.

Among vaccinated children, we examined the timing of varicella vaccine administration. The first dose was administered at a median age of 12 months (interquartile range, 12–13), in accordance with the recommended schedule. The mean age at first dose was approximately 13 months among 1-dose recipients and approximately 12.8 months among children who later received 2-dose, indicating that most infants received varicella vaccination on schedule around their first birthday. Among children who received a second dose, the median age at administration was 53 months (approximately 4 years and 5 months). The mean±standard deviation age at second dose was approximately 59±15 months, with most second doses administered between 4 years and 6 years of age.

Two main varicella vaccine strain lineages were used in Korea during the 2011–2022 period: the Oka strain and the MAV strain. Table 2 presents the distribution of vaccine strains administered within the cohort. For the first dose, children were approximately equally likely to receive an Oka-strain vaccine or an MAV-strain vaccine. Among 1-dose recipients, 52.6% received an Oka-strain vaccine and 46.9% received an MAV-strain vaccine. A similarly balanced distribution was observed for the first dose among children who ultimately received second doses, with approximately 44.1% receiving an Oka-strain vaccine and 54.9% receiving an MAV-strain vaccine.

Varicella vaccinations in Korea are administered across a range of healthcare settings, and the NIP permits private providers to administer vaccines at no cost to patients. In this cohort, the majority of varicella vaccine doses were administered in private community clinics, followed by hospitals, with a smaller proportion delivered through public health centers. Specifically, approximately 64.8% of first-dose vaccinations occurred in community clinics, and an additional 24.0% were administered in hospitals. Public health centers, which are government-run facilities, accounted for 8.9% of first doses. The remaining first doses were administered in other facility types or were uncategorized, collectively accounting for less than 2.3%. For second doses, an even larger proportion (86.3%) was administered in clinics, with 12.8% given in hospitals and approximately 0.1% in public health centers.

### Patient and public involvement

Patients and members of the public were not involved in the design, conduct, or reporting of the K-VIM Scheme. The cohort was derived entirely from registry and insurance claims data, and no participant recruitment or primary data collection was performed. Study findings will be disseminated in aggregated form to health authorities and the public; however, there was no active patient or public involvement in setting the research agenda.

### Ethics statement

This study involves human participants, and the conduct of this study was reviewed and approved by the Institutional Review Board (IRB) of Korea University Ansan Hospital (IRB No. 2021AS0127).

## KEY FINDINGS

### Findings to date

Although the primary objective of the K-VIM Scheme is longitudinal monitoring, baseline analyses revealed notable patterns in varicella incidence according to vaccination status.

Table 3 presents the crude cumulative incidence of varicella across vaccination groups. It is critical to emphasize that these values represent crude proportions rather than person-time–based incidence rates. The groups are not directly comparable due to substantial confounding by age and follow-up duration. As shown in Table 2, the unvaccinated group consists predominantly of infants born in 2022, who contributed minimal follow-up time, explaining their low observed crude incidence. These crude figures should not be interpreted as estimates of vaccine effectiveness.

For the purposes of this descriptive analysis, children were classified according to their final vaccination status as of December 31, 2022. Varicella cases that occurred after receipt of the first dose but prior to administration of a second dose were attributed to the 1-dose group.

Among the 4.5 million children included in the cohort, a total of 287,216 varicella cases were identified through 2022. Of these, 24,988 children who received varicella vaccination after experiencing varicella and 7,360 children who developed varicella within 42 days of vaccination were excluded, resulting in a total of 32,348 excluded individuals. When stratified by vaccination status, varicella infections were most frequently observed among children who had received only 1 vaccine dose. Approximately 9.09% of 1-dose recipients developed varicella by the end of follow-up, compared with 1.28% of children who received 2-dose.

A portion of these analyses has been published previously in a peer-reviewed journal [15]. That study evaluated the effectiveness of 1-dose and 2-dose varicella vaccination in Korea using a national cohort and a matched case-control design.

## STRENGTH AND LIMITATIONS

The K-VIM cohort is one of the largest and most comprehensive population-based varicella studies conducted to date, encompassing more than 4.5 million children nationwide. By leveraging linked administrative databases, the cohort effectively functions as a whole-population longitudinal study with minimal loss to follow-up. A major strength is the complete capture of vaccination records through the national immunization registry. Because varicella vaccination is universally recommended and systematically recorded, we have high confidence in the ascertainment of vaccination status, timing, and vaccine product. Similarly, varicella outcomes were identified using comprehensive insurance claims data from the HIRA. We consider the accuracy of these claims to be high, as varicella is also a nationally notifiable disease, which incentivizes accurate clinical diagnosis by physicians. Another important strength is the ability to link multiple data sources at the individual level, enabling adjustment for confounding variables and detailed characterization of cases. Finally, the reliance on existing electronic data renders the cohort cost-efficient and allows for regular updates, facilitating near real-time monitoring of epidemiological trends.

Despite these strengths, several limitations are inherent to the use of administrative data for epidemiological research. First, because nearly all Korean children receive varicella vaccination, the unvaccinated group constitutes a small and potentially non-random subset of the population. The cohort contains key variables that permit adjustment in future analytic studies, including birth year, sex, region, and healthcare utilization frequency, although such adjustments were not applied to the descriptive analyses presented in this profile. Second, overall varicella vaccine coverage is very high, exceeding 93% for at least 1 dose, which limits statistical power for comparisons between vaccinated and unvaccinated children. Moreover, children who remain unvaccinated may not be representative of the general pediatric population. Third, varicella outcomes were identified using diagnostic codes from insurance claims data (ICD-10 code B01.x), without laboratory confirmation. A formal validation study of this diagnostic code was not conducted for this cohort, representing an important limitation. We also did not cross-validate outcomes with data from the national notifiable disease surveillance system. However, because varicella is a nationally notifiable disease and presents with a distinctive clinical rash, we anticipate that the specificity of physician-assigned diagnostic codes is high, although some degree of misclassification remains possible. Fourth, because case ascertainment relies on healthcare utilization records, varicella cases among children who did not seek medical care would not be captured. Given that healthcare coverage in Korea is universal and affordable, most families are likely to seek medical attention for varicella, but asymptomatic or very mild cases may have gone undetected. Consequently, incidence may be modestly underestimated, and children who never accessed healthcare services would appear disease-free in the data. Fifth, there may be incomplete documentation of second-dose vaccinations administered outside the NIP. For example, if a child received a second dose abroad or in a setting where vaccination was not recorded in the registry, that child would be misclassified as having received only 1-dose. Additionally, we cannot distinguish the clinical indications for 2-dose vaccination. Although many children received a second dose as a privately funded supplemental vaccination, we cannot reliably identify specific high-risk subgroups, such as immunocompromised children, who may have followed alternative vaccination schedules. This limitation restricts subgroup-specific analyses. Furthermore, the K-VIM monitoring system currently relies on passive surveillance using diagnostic codes from insurance claims. Incorporation of active surveillance components in the future could strengthen case ascertainment. Finally, as an observational study, causal inference must be undertaken cautiously. The crude incidence differences presented in Table 3 should not be interpreted without accounting for the potential biases discussed above, and analytic study designs are required to estimate vaccine effectiveness appropriately.

In summary, the K-VIM Scheme demonstrates the feasibility of large-scale data linkage studies for vaccine impact assessment in Korea. By harmonizing immunization records and health outcome data at the individual level, this cohort provides a powerful resource for public health research and policy decision-making. Continued refinement of data quality and analytic approaches will further strengthen evidence regarding varicella vaccine performance. Despite its limitations, the breadth and depth of the K-VIM cohort position it as a cornerstone resource for varicella epidemiology and immunization program evaluation in Korea.

### Future plans

The K-VIM cohort constitutes a critical resource for investigating the long-term impact of Korea’s varicella vaccination program. Future studies will extend beyond varicella incidence to evaluate potential long-term effects on herpes zoster incidence as vaccinated cohorts age. A key priority will be the analysis of temporal trends through stratification of vaccine coverage and breakthrough infection incidence by birth cohort. Such analyses will demonstrate the longitudinal analytic capacity of the dataset and clarify how the program’s impact has evolved over time.

In addition, future investigations must address the methodological challenges posed by the small and non-representative unvaccinated group. To enhance internal validity and minimize selection bias, analytic strategies will prioritize robust comparisons between 1-dose and 2-dose recipients, which represent a central policy question in light of concerns regarding waning immunity following a single dose. Rigorous methods, including propensity score matching, will be employed to construct comparable study groups and strengthen causal inference.

### Collaboration

Initial data analyses and publications will be conducted by the study investigators. The research team welcomes potential research collaborations. Investigators interested in collaboration should contact the corresponding author. Access to data and analytical files is subject to approval by the relevant research ethics committees and data custodians. Owing to ethical and regulatory considerations, analysis of linked data is currently authorized at a single secure location.

## Figures and Tables

**Table 1.** Enrollment by birth cohort

Birth year	Male	Female	Total
2011	244,291 (5.4)	231,709 (5.1)	476,000 (10.6)
2012	251,119 (5.6)	238,230 (5.3)	489,349 (10.9)
2013	225,900 (5.0)	215,098 (4.8)	440,998 (9.8)
2014	225,516 (5.0)	214,740 (4.8)	440,256 (9.8)
2015	227,669 (5.1)	216,460 (4.8)	444,129 (9.9)
2016	211,157 (4.7)	201,538 (4.5)	412,695 (9.2)
2017	187,387 (4.2)	176,641 (3.9)	364,028 (8.1)
2018	170,893 (3.8)	162,242 (3.6)	333,135 (7.4)
2019	158,421 (3.5)	150,017 (3.3)	308,438 (6.9)
2020	142,414 (3.2)	135,893 (3.0)	278,307 (6.2)
2021	136,346 (3.0)	129,513 (2.9)	265,859 (5.9)
2022	128,934 (2.9)	123,037 (2.7)	251,971 (5.6)
Total	2,310,047 (51.3)	2,195,118 (48.7)	4,505,165 (100)

Values are presented as number (%).

**Table 2.** Characteristics of varicella vaccine groups

Characteristics	Unvaccinated group (n=298,533)	1-dose vaccinated group (n=2,852,547)	2-dose vaccinated group (n=1,321,737)	p-value
Birth year				<0.001
2011	12,603 (4.2)	320,191 (11.2)	137,486 (10.4)	
2012	12,139 (4.1)	305,579 (10.7)	166,329 (12.6)	
2013	10,768 (3.6)	257,051 (9.0)	168,909 (12.8)	
2014	10,479 (3.5)	236,514 (8.3)	189,549 (14.3)	
2015	10,456 (3.5)	223,951 (7.9)	206,238 (15.6)	
2016	9,833 (3.3)	205,996 (7.2)	193,849 (14.7)	
2017	9,723 (3.3)	209,185 (7.3)	142,993 (10.8)	
2018	9,663 (3.2)	224,777 (7.9)	96,890 (7.3)	
2019	8,544 (2.9)	280,966 (9.9)	17,797 (1.4)	
2020	8,188 (2.7)	267,826 (9.4)	1,387 (0.1)	
2021	11,433 (3.8)	253,336 (8.9)	305 (0.1)	
2022	184,704 (61.9)	67,175 (2.4)	5 (0.1)	
Sex				<0.001
Male	152,413 (51.1)	1,471,123 (51.6)	669,354 (50.6)	
Female	146,120 (48.9)	1,381,424 (48.4)	652,383 (49.4)	
Age of first dose vaccine (mo)				<0.001
Mean±SD	–	13.17±3.96	12.81±2.26	
Median (range)	–	12 (12–13)	12 (12–13)	
Age of second dose vaccine (mo)				<0.001
Mean±SD	–	–	58.68±14.79	
Median (range)	–	–	53 (49–68)	
Vaccine strain				<0.001
First dose vaccination				
Oka	–	1,499,051 (52.6)	583,260 (44.1)	
MAV	–	1,337,612 (46.9)	726,044 (54.9)	
Unknown	–	15,884 (0.6)	12,433 (0.9)	
Second dose vaccination				
Oka	–	–	808,187 (61.2)	
MAV	–	–	494,841 (37.4)	
Unknown	–	–	18,709 (1.4)	
Vaccination facility				<0.001
First dose vaccination				
Hospital	–	684,882 (24.0)	275,371 (20.8)	
Clinic	–	1,848,432 (64.8)	933,524 (70.6)	
Public health center	–	252,685 (8.9)	71,310 (5.4)	
Others	–	356 (0.1)	234 (0.1)	
Unknown	–	66,192 (2.3)	41,298 (3.1)	
Second dose vaccination				
Hospital	–	–	169,666 (12.8)	
Clinic	–	–	1,141,206 (86.3)	
Public health center	–	–	1,043 (0.1)	
Others	–	–	450 (0.1)	
Unknown	–	–	9,372 (0.7)	

Values are presented as number (%). SD, standard deviation.

**Table 3.** Comparison of the incidence of varicella by vaccine status

Variables	Unvaccinated group (n=298,533)	1-dose vaccinated group (n=2,852,547)	2-dose vaccinated group (n=1,321,737)	p-value
Breakthrough varicella				<0.001
No	287,617 (96.3)	2,593,161 (90.9)	1,304,823 (98.7)	
Yes	10,916 (3.7)	259,386 (9.1)	16,914 (1.3)	
Type of treatment				<0.001
Outpatient	10,158 (93.1)	255,496 (98.5)	16,795 (99.3)	
Inpatient	758 (6.9)	3,890 (1.5)	119 (0.7)	
Age of diagnosis (mo)	17.53±20.52	53.96±24.23	79.28±18.61	<0.001

Values are presented as number (%) or mean±standard deviation.

## Data Availability

Data may be obtained from a third party and are not publicly available. Direct access to the data and analytical files is not permitted without the express permission of the approving human research ethics committees and data custodians. Researchers interested in collaboration should contact the corresponding author.
